# Goldbaum

**Published:** 1885-07-20

**Authors:** 


**Goldbaum** recommends burnt alum as an application in most instances where iodoform is used as an antiseptic. In slight wounds he thinks the alum all sufficient, but in certain cases would mix the two drugs in the proportion of 2 parts of aluminis ustæ to 1 part of iodoform. Vratsch.

